# Consensual Regression of Soluble Solids Content in Peach by Near Infrared Spectrocopy

**DOI:** 10.3390/foods11081095

**Published:** 2022-04-11

**Authors:** Lei-Ming Yuan, Lifan You, Xiaofeng Yang, Xiaojing Chen, Guangzao Huang, Xi Chen, Wen Shi, Yiye Sun

**Affiliations:** College of Electrical & Electronic Engineering, Wenzhou University, Wenzhou 325035, China; yuan@wzu.edu.cn (L.-M.Y.); lifanyou8@foxmail.com (L.Y.); yxf1628615810@163.com (X.Y.); chenxj@wzu.edu.cn (X.C.); cx@wzu.edu.cn (X.C.); shiwen@wzu.edu.cn (W.S.); syy@wzu.edu.cn (Y.S.)

**Keywords:** peach, near-infrared spectroscopy, genetic algorithm, partial least squares, consensus fusion

## Abstract

In order to reduce the uncertainty of the genetic algorithm (GA) in optimizing the near-infrared spectral calibration model and avoid the loss of spectral information of the unselected variables, a strategy of fusing consensus models is proposed to measure the soluble solids content (SSC) in peaches. A total of 266 peach samples were collected at four arrivals, and their interactance spectra were scanned by an integrated analyzer prototype, and then an internal index of SSC was destructively measured by the standard refractometry method. The near-infrared spectra were pre-processed with mean centering and were selected successively with a genetic algorithm (GA) to construct the consensus model, which was integrated with two member models with optimized weightings. One was the conventional partial least square (PLS) optimized with GA selected variables (PLS_GA_), and the other one was the derived PLS developed with residual variables after GA selections (PLS_RV_). The performance of PLS_RV_ models showed some useful spectral information related to peaches’ SSC and someone performed close to the full-spectral-based PLS model. Among these 10 runs, consensus models obtained a lower root mean squared errors of prediction (RMSEP), with an average of 1.106% and standard deviation (SD) of 0.0068, and performed better than that of the optimized PLS_GA_ models, which achieved a RMSEP of average 1.116% with SD of 0.0097. It can be concluded that the application of fusion strategy can reduce the fluctuation uncertainty of a model optimized by genetic algorithm, fulfill the utilization of the spectral information amount, and realize the rapid detection of the internal quality of the peach.

## 1. Introduction

Peaches, belonging to the variety of *Prunus persica,* contain a unique taste, flavor, sweetness and texture. They are rich in vitamin C, carotene, pectin, and many kinds of trace elements such as zinc and selenium [1], and are widely welcomed by a broad range of ages. With the upgrade of consumption and living quality, consumers pay more attention to the internal quality of fruit, not just the external. Soluble solids content (SSC) is an important indicator of maturity and is commonly used to estimate the internal quality of a peach. The degree of SSC highly relates to the sensory and acceptance of consumers, and further influences the shelf-life price of fruit [2,3]. Obviously, SSC plays an important role to improve the competitiveness of fruit products and market economic value.

Currently, determination of peaches’ SSC mainly depends on the destructive refractometry detection method, it can obtain high precision, but it destroys the integrity of fruit samples, causing damage of fruit and affecting secondary sales. At the same time, the speed of the refractometry method is just for random determination, and thus it is not suitable to detect high-throughput samples [4]. Therefore, the realization of a simple and rapid non-destructive determining technology for the internal quality of peaches, can not only improve the market economic value of peach fruit, but also standardize the management of the peach market and promote the income of fruit farmers, which has important guidance for the industrial upgrading of the fruit market [5,6,7].

As an instrumental analytical technique, near infrared spectroscopy (NIRS) is well-known in sensing components of material [8,9]. Its major benefit is a non-destructive method, and usually just a simple, or no preparation, needs to be performed. It can yield an online response for analysis during manufacturing, being rapid, non-invasive, very flexible and robust. NIRS technology has been reportedly widely used in food, agriculture and medical areas [4,5,10], especially in the rapid detection of fruit internal quality, such as pear [11], orange [12], apple [13,14]. In order to replace the destructive refractometry detection method, it is essential to guarantee accurate predictions by the application of NIRS technology. For modeling the relationship between spectral data and quality attributes, classical statistical methods of multivariate analysis, such as multiple linear regression (MLR), principal component regression (PCR) and partial least squares (PLS) have to be considered.

However, visible-near infrared spectra (Vis-NIR) usually contains hundreds of spectral variables, which not only contain useful information, but also involve a variety of invalid information, and there exists the co-linear problem between neighboring spectral variables [15,16]. It is therefore necessary to employ the screening methods aimed at reducing the dimension of spectra. Many variable selections have been proposed to select the informative variables and get the performance of model improved [17], such as competitive adaptive re-weighted sampling (CARS), successive projections algorithm (SPA), uninformative variable elimination (UVE), simulated annealing (SA), and genetic algorithm (GA). But some selection methods enhance the predictive ability of models, and meanwhile increase the uncertainty of variable selection, including the number and the selected variables and their combinations, such as GA, which is proposed on the basis of evolutionary theory, that the ‘best’ individuals (i.e., wavelengths or variables) have a better chance to survive and a larger probability to spread their genomes by reproduction in a living system [16,17,18,19,20].

At present, most modeling methods adopt a single or uni-vocal model to quantitatively predict the quality of fruit. One single model can overcome some kind of disturbance factor, but it can not avoid the influences of many other disturbance factors [21,22]. Those above variable selection methods can go through certain rules to obtain the best combination of useful variables, so as to make the model achieved of the best predictive performance. However, this commonly intends to overcome the interference of one specific factor. As for the GA method, the combination of the ‘best’ individuals varies from the initial genomes, and thus leads to a different number of variables and different spectral wavelengths. When GA is used to optimize the spectral model, the combination of the selected variables is differently varied from each running. This is going to increase the uncertainty of the result by the operation of GA. Besides, is there any useful information among the remaining variables? This should be explored. The full spectral variables involved in the model usually contain some redundant and irrelevant information, which complicates the model and reduces the prediction accuracy of the model. With utilization of GA variable selection, the performance of the calibration model can be enhanced, but results in uncertainty of the combination of the selected variables and the loss of information in residual variables, and different individuals are likely to lead to different results [17,23,24].

To solve these problems, in this work the fusing strategy of the consensus model was proposed to combine the GA variable selection algorithm at the decision level of the member models, aiming to improve the prediction accuracy and reduce the uncertainly of the model [17,22,25,26,27]. The regression member models were developed between the main indicator SSC of peaches and their interactive spectra. Member models were used to construct the consensus model through arranging the weightings according to their performances. One was the optimized model PLS_GA_, which was developed with the selected variables by the GA method, and another was the PLS_RV_ model, which was developed with the residual variables that were not selected in the above GA running. It should be noted that more batches and orchards of peaches harvested with different degrees of maturity using vis-NIR spectroscopy need to be investigated, and thus the applicability of the developed model should be robust and achieve generalized feasibility.

## 2. Materials and Methods

### 2.1. Sample Preparation

The bagging juicy peaches of cultivar Xinchuanzhongdao were harvested at the period of harvestion at the end of July 2020 in Wenzhou city, Zhejiang province, China. Peach samples were collected every other day and, in total, four batches of peaches were arranged in this work. After transporting to the lab, peaches were unbagged to discard improper samples by technicians, and a total of 266 samples were selected without diseases, pests and mechanical damage et al., and were stored in an air-conditioned room of 22 °C for at least 6 h. The range of equatorial diameter of these peaches was in 45~75 mm and the weights were in between 110~330 g. Samples were orderly numbered and three sites were marked on samples’ equatorial line with equal interval, for subsequent measurements of spectral signal and reference value.

### 2.2. Spectral Acquisition

**Figure 1:**
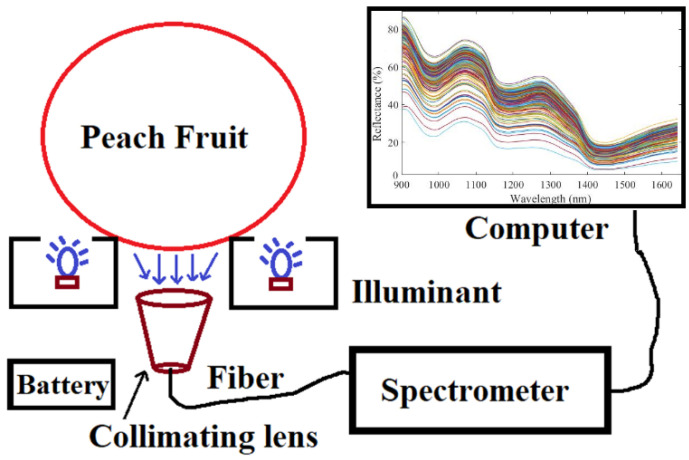
Schematic diagram of peach spectrum acquisition.

Interactance spectra of peaches are collected by an integrated portable NIR analyzer (Figure 1), which is embedded with a commercial spectrometer (Model: flame-NIR, ocean Optics Inc., Dunedin, FL, USA), battery module, halogen sources (MR11, 12V 20W, Orsam) and a soft gasket holder for supporting the peach sample. Four halogen light sources are arranged symmetrically through the light channels upward to the sample’s holder. A soft silicone gasket is attached to the holder (with a diameter range of 10~15 mm), and thus it not only prevents the sample from moving, but also minimizes the interference of external light into the detector. The local penetrating signal of peach is filtered by a collimating lens and through the optical fiber transferring into the entrance of the flame-NIR spectrometer. The scanning band range of the spectrometer is 902.59~1648.61 nm with a resolution of 20.0 nm, and the number of spectral wavelengths is 227. The scanning parameter is set as the integration time of 0.2 s, a smoothing window of size 3, and the average scanning number of 4. In this experiment, spectral data are recorded from three different sites of each peach, and then the average spectrum is calculated as the final spectral curve of each peach sample.

### 2.3. Measurement of Soluble Solids Content

A digital refractometer PAL-1 (Atago Co., Ltd., Tokyo, Japan) is used to measure the soluble solids content (SSC) of peach with a precision of ±0.1% Brix. After peeling, the pulp is obtained around three marked sites (i.e., the spectral reading point), and mixed to squeeze into juice. The juice is measured on the digital refractometer. This process was repeated three times, and their values were averaged as SSC value for the peach sample.

### 2.4. Multivariable Data Analysis

Pretreatments, including the first derivation with Savitzky–Golay smoothly moving, using five points of second polynomial order (S-G D1st), multiplicative scatter correction (MSC), mean centering (MC) and standard normal variate (SNV), are employed to improve the quality of spectra and promote the ratio of signal to noise.

GA is used to select the “best” individuals (i.e., spectral variables) that have a greater chance of surviving and a higher probability to pass on their genomes by the reproduction of evolutionary theory [19]. There are five primary steps contained in the spectral variables’ selection, and they are: variable encoding, population initiation, response evaluation, reproductions, and population. In the stage of the first two steps, the encoded genomes are varied, and thus the result of each GA’s operation is changed. Therefore, usually more than five runs are performed on the spectral data to select the optimized combination of spectral variables [13].

Partial least square (PLS) is used to develop a quantitative model between spectral data and peaches’ attributes. Spectra are above the “best” individuals selected by the GA program, and are mapped into an orthogonal linear space, where the top several latent variables (LVs) accumulate useful spectral information, and the number of LVs in the PLS model is determined by the smallest RMSECV in the calibrating stage and considered as the optimal mappings corresponding to fit attributes [28].

From the view of the fusing level [26], in this work, the decision level of fusing strategy is adopted to construct the consensus model, which integrates several member models, rather than one single model. Based on the consensual rule, two or more member models are assigned with different weighting coefficients according to the significant degree of member models [11,25]. It can reduce the dependence of a single model to weaken the influence of some specific correlated factors. Its mathematical expression principle is that: (1) consensus model *F*(*x*) is expressed as the linear combination (Equation (1)) of *n* member models with weightings of *w_k_*; (2) the constraint conditions are required the minimization of summed residuals squares, and the weightings *w_k_* in the range of 0~1, and their accumulation equals to 1 (Equation (2)); (3) the inferred surplus of $\mathrm{ARG}\min(\sum_{k=1}^{n}{\left(w_{k}\cdot e_{k}\right)}^{2}$ is solved by the Lagrange multiplier method [25], where *e_k_* was the predicted residual of the *k*th member model.

(1) $$\left\{ \begin{array}{l} F(x)=\sum_{k=1}^{n}w_{k}f_{k}(x)\\ ARG\min{\langle y-F(x)\rangle }^{2} \end{array}\right.$$

(2) $$s._{}t_{}\left\{ \begin{array}{l} 0\leq w_{k}\leq 1\\ \sum w_{k}=1 \end{array}\right.$$

where $\sum_{k=1}^{n}{\left(w_{k}\cdot e_{k}\right)}^{2}$ is inferred from Equation (3), and its error $E(e^{2})$ can be calculated as further expansion of Equation (4).

(3) $$ARG\min{\left(y-F(x)\right)}^{2}=\sum_{k=1}^{n}{\left(w_{k}(y-f_{k}(x_{k}))\right)}^{2}=\sum_{k=1}^{n}{\left(w_{k}\cdot e_{k}\right)}^{2}$$

(4) $$E(e^{2})=\sum_{k=1}^{n}w_{k}{}^{2}e_{k}{}^{2}+2\sum_{k=1}^{n}\sum_{j>i}^{n}w_{k}w_{j}e_{k}e_{j}=\sum_{k=1}^{n}w_{k}{}^{2}e_{k}{}^{2}+\sum_{i=1}^{n}\sum_{j>i}^{n}w_{k}w_{j}r_{kj}e_{k}{}^{2}$$

It is assumed that the predicted deviation ***e_k_*** obeys the normal distribution $N(0,\sigma^{2})$, and represents the ignored random factors in the *k*-th member model. These random factors are assumed to be independent of each in member models, and thus the array of {*e_1_*, *e_2_*, …, *e_n_*}, as well as the final predicted deviation ***e*** in the developed consensus model should approximately obey the normal distribution. Thus, the impacts of error vector correlation in member models can be ignored, and $\sum w_{kj}\cdot e_{k}{}^{2}=0$ in Equation (4) can be assumed.

All calculations in this study were performed in the MATLAB software (R2018a, Math Works Inc., Natick, MA, USA). The PLS algorithm was performed using the iToolbox [28]. The fusion codes were programed referring to the above formulas.

## 3. Results

### 3.1. Distribution of SSC

**Figure 2:**
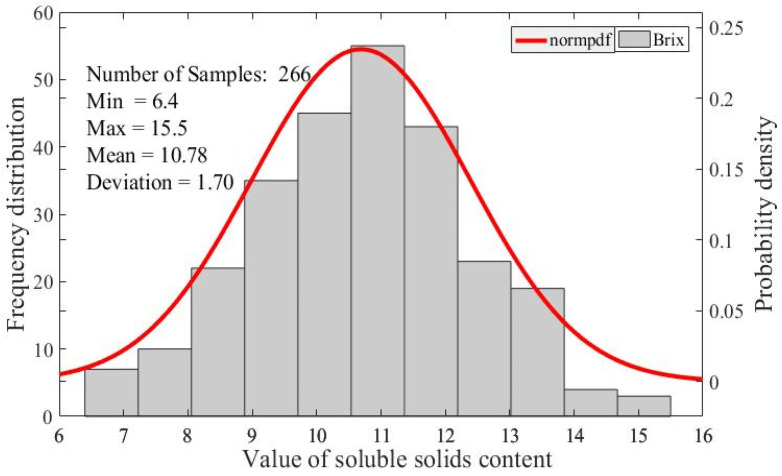
Spectrum of soluble matter content in peach samples.

The histogram of peaches’ SSC is shown in Figure 2. Values of SSC in these 266 samples are distributed normally in the range of 6.4 ~15.5%, and the average value is 10.89% with a standard deviation of 1.7%. The range of the ‘Xinchuanzhongdao’ cultivar’s SSC measured in this research is similar to that of ‘Hongmi’ cultivar [29] and ‘Aurora-1’ cultivar peach fruit [30], indicating the random harvest of sample fruits with a small difference of SSC between peaches’ cultivars. It also observes that during the period of harvest the maturity of peaches is in a broad range, inferred by the distribution of peaches’ SSC.

A total of 266 samples were divided into two subsets with the ratio of 2:1 by a typical duplex method as shown in Table 1. One is the calibration subset, used to construct and train the calibration regression model, and the other one is the prediction subset, used to validate the feasibility of the developed regression model. The mean of SSC values in these two subsets are close, indicating that the homogeneous distribution of divisions is made to evenly develop the regression model.

### 3.2. Spectral Analysis and Pretreatment

**Figure 3:**
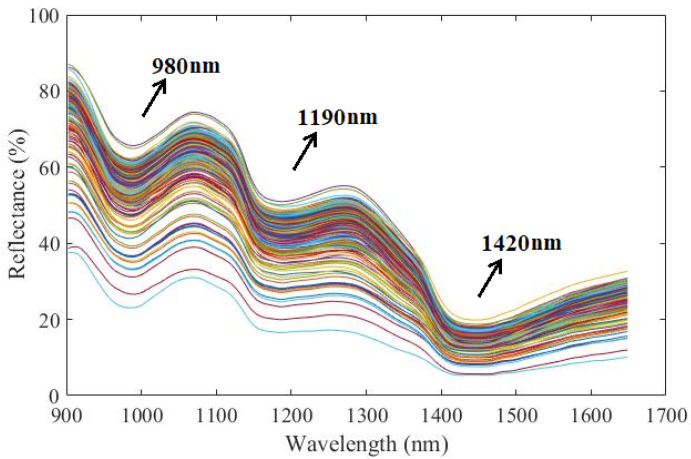
Original near infrared reflectance spectra of peaches. One curve stands for one sample. Spectral curves with different colors are convenient for readers to view the trend of spectra.

Figure 3 shows the original near infrared reflectance spectra of 266 peach samples, whose spectral tendency are consistent but with differences of spectral intensity. There are several valleys, mainly around at 980 nm, 1190 nm, and 1420 nm involved in the peach’s spectrum, indicating the absorption of energy by special functional groups of molecules [31]. The valley at 980 nm is referred to associate with the second overtone of the O-H group. The valley at 1190 nm is related to the combination of C-H stretching, C-O stretching and O-H stretching groups in some macro-molecular substances, such as cellulose, pectin and starch. The strong absorption valley at 1420 nm is due to the first overtone of N-H stretching and the first overtone of O-H stretching groups, mainly caused by the 85–95% moisture in the intact peach fruit [32]. Obviously, the spectral absorptions are correlated to the functional groups of samples’ attributes by the naked-eye, but the concentration value of attributes could not be given out through direct observation of the NIR spectral profile due to its severely overlapped information and the multivariate data modeling analysis needed for prediction.

To enhance the spectral efficient information and promote the performance of PLS calibration models, four different spectral pretreatments were employed to process the original spectra, and then the pre-processed spectra in the calibration subset were fully used to construct the PLS model, with cross validation in optimizing the number of latent variables (LVs). Table 2 shows the statistical results of the developed PLS models’ performances in predicting the SSC of peaches. By comparison of parameters RMSE, *r* and *Bias* in these models, corresponding to pretreatments of SNV, MSC and MC, the PLS model based on the full pre-processed spectra obtained better performances than those without any pre-processed method, except that the performance of the PLS model with S-G D1^st^ pretreatment was worse. It may be explained that the differential operation not only removes the uninformative background signals, but also magnifies local noise involved in the spectra. The PLS model with MC pre-process had the best performance than any others, providing RMSECV of 1.017%brix in the cross validation stage, and RMSEP of 1.129%brix in the prediction stage. Clearly, MC can improve the ratio of signal to noise in the original spectra and reduce variations between spectra of multiple batches of peaches [22], concerned on enhancing the predictive ability of the developed PLS model, reducing the RMSEP by 1.14%.

### 3.3. Variables Selected by GA Method

Since the above full spectral wavelengths are used to construct the quantitative PLS model, which comprises some redundant and useless variable information, this may compromise the predictive accuracy of the model. In this study, the commonly used genetic algorithm (GA) is adopted on the MC pre-processed spectra and the variables selected by the GA method are used to develop the PLS model (PLS_GA_, labelled as *f_i_*_1_), while the residual variables (that are unselected) are also used to develop the PLS model (PLS_RV_, labelled as *f_i_*_2_). Due to the random encoding of spectral wavelengths, 10-time runs of the GA method (more than 30 runs are taken out) are carried out successively, and the selected and the residual spectral variables are recorded for subsequent modeling.

Table 3 shows the statistical results of PLS member models’ performances in predicting the SSC of peaches by optimization of the GA method. Compared with the full spectral-based PLS model, the predictive performances of PLS_GA_ models are improved by less spectral variables. Parameter *R_cv_* of PLS_GA_ models are in the range of 0.811~0.832, and are clearly higher than that of the previous full spectral-based PLS model. RMSECV are all reduced and in the range of 0.9~0.954%. Compared to the original full spectral-based PLS model, the averaged RMSECV in these 10 PLS_GA_ models reduces from 10.1 percent to 0.926%, and in terms of predicting external samples, the RMSEP averagely reduces 2.3 percent to 1.116%. Among these optimized PLS_GA_ models, the 6th and 9th PLS_GA_ models are performed better than others. Meanwhile, just a small number of spectral variables are selected to develop these calibration models, and their performances get better than that of the original PLS model. The above shows that the GA method can reduce partial interference or useless information, and enhance the predictive captivity of the regression model.

Taking a close observation on Table 3, PLS_RV_ models that are developed with the residual variables performed not worse, and some are closed to the original PLS model with the RMSECV in these PLS_RV_ models ranging from 1.055~1.096%. In terms of predicting, some PLS_RV_ models also performed well on the external samples. What is interesting is that the residual variables, not selected as the “best individuals” in the routine of GA processing, also comprise some useful spectral wavelengths through modeling. It can be said that PLS_RV_ models developed with the residual variables can achieve nearly approximate performance as the full-spectra-based models.

### 3.4. Fusion of Member Models

**Figure 4:**
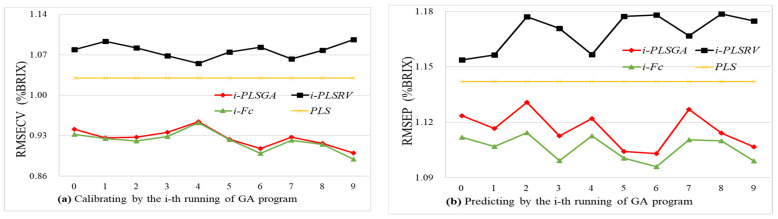
Root mean square error of models with the selected variables by GA, residual variables, and consensus fusion (**a**) Calibration set (**b**) Prediction set.

In order to make full use of the information from spectra, and to further improve the performance of the calibration model, the consensual regression model (*F_c_*) was proposed to integrate above two regression models, and they were PLS_GA_ model based on GA selected variables and PLS_RV_ models based on residual variable through GA runs, respectively. Thus, the *i*-th consensual model (*F_ic_*) was constructed based on the *i*-th PLS_GA_ (*f_i_*_1_) and the *i*-th PLS_RV_ (*f_i_*_2_) by the formula Equations (1) and (2) at the period of *i*-th running of the GA program, and a total of 10 *F_c_* models were obtained. Then, samples in the calibration set and prediction set were put into each consensual model, and parameters of prediction were counted, and are shown in Figure 4.

It can be seen from Figure 4a that the root mean squared error of cross-validation (i.e., RMSECV) in the calibration set by the consensus model (*F_c_*) is close to or slightly lower than that of the corresponding PLS_GA_ model (*f_i_*_1_), and the tendency of these two models’ performances are consistently validated in the calibration set. However, it turns out to be completely different in the prediction stage. The consensus model obtained the average RMSEP of 1.106% with a standard deviation of 0.0068, while the optimized PLS_GA_ model achieved the averaged RMSEP of 1.116% with a standard deviation of 0.0097. In Figure 4b, each consensus model (*F_c_*) performed better than the optimized PLS_GA_ model (*f_i_*_1_) in predicting the prediction set, and their performances (*F_c_*) were promoted with an average of 2.27% in the range of 0.98~3.42% in the calibration set, and were enhanced an average of 3.14% in the range of 2.57~4.03% in the prediction set compared to the original PLS model. Among these, the *F_6c_* consensual model reduced RMSRP to 1.096% with the highest improvement of predictive capacity. Obviously, consensual models among these developed models trended to be more stable with small fluctuations in the prediction stage.

Among these series of continuous running PLS_GA_, PLS_RV_, and consensual models, overall, PLS_GA_ performed better than PLS_RV_, and meanwhile the consensual model performed better than PLS_GA._ In rare cases, concerning the prediction stage, the PLS_RV_ model performed approximately to the PLS_GA_ model and the full-spectral-based PLS model. On the one hand, although “the best individuals” useful variables are filtered out from the full spectra by the GA method, the residual spectral variables still contain some that can reflect the internal quality of peach fruit. On the other hand, a genetic algorithm is not deterministic to construct PLS model, but is a relatively well-behaved approach to optimize the combination of spectral variables.

To sum up, the consensual modeling approach makes full use of the spectral information in avoiding the loss of remaining spectral variables, and fuses member models into a consensual measurement on highlighting the individuality of member models and compressing their commonality, and thus to improve the prediction performance of consensual models, and avoid the uncertainty caused by genetic algorithms or other variable selection methods.

## 4. Conclusions

The internal quality of peach was rapidly detected by a portable device integrated with a near-infrared spectrometer, and a consensual measurement based on multi- member models was proposed to predict the SSC of peach. The residual variables after GA selections still provided the spectral information correlated with peaches’ interiors, and the consensus model performed better than the PLS_GA_ model, and lowered the RMSEP with an average of 3.14% compared to the original PLS model. This proposed fusing method can be applied with other variable selections, such as SA and UVE, to avoid the uncertainty of the model and loss of spectral information, and improve the stability of the model.

## Figures and Tables

**Table 1:** Distribution of soluble solids content in peach samples.

	Number	Max	Min	Mean	Standard Deviation
Calibration set	177	15.0	6.4	10.61	1.63
Prediction set	89	15.5	6.4	10.86	1.83

**Table 2:** Comparison of pretreatments on the predictive performance of the developed PLS model.

Pretreatments	LVs	Calibration Set			Prediction Set		
		*RMSECV*	*R_cv_*	*Bias*	*RMSEP*	*R_p_*	*Bias*
S-G D1st	9	1.066	0.755	−0.030	1.297	0.733	−0.015
MSC	9	1.029	0.779	−0.005	1.139	0.765	−0.006
SNV	9	1.029	0.778	−0.008	1.141	0.764	−0.010
MC	11	1.017	0.792	−0.002	1.129	0.771	−0.008
None	10	1.030	0.773	−0.003	1.142	0.762	−0.007

Note: MSC: Multiplicative Scatter Correction; SNV: Standard Normal Variate; MC: mean centering; S-G D1st: First deviation with S-G smoothing; PLS: partial least squares;.

**Table 3:** Predictive performance of PLS models by genetic algorithm method.

Member Model	Selected/Residual Variables	Calibration Subset		Prediction Subset	
		*RMSECV*	*R_cv_*	*RMSEP*	*R_p_*
***f*_01_**	62 ^a^ (6) ^b^	0.942	0.817	1.124	0.786
***f*_02_**	165 (8)	1.079	0.735	1.154	0.723
***f*_11_**	55 (5)	0.926	0.823	1.117	0.787
***f*_12_**	172 (6)	1.0931	0.715	1.157	0.725
***f*_21_**	35 (5)	0.928	0.821	1.131	0.780
***f*_22_**	192 (7)	1.082	0.735	1.177	0.721
***f*_31_**	33 (4)	0.936	0.819	1.113	0.784
***f*_32_**	194 (8)	1.068	0.736	1.171	0.725
***f*_41_**	52 (5)	0.954	0.811	1.122	0.783
***f*_42_**	175 (7)	1.055	0.738	1.157	0.716
***f*_51_**	43 (4)	0.924	0.823	1.104	0.791
***f*_52_**	184 (7)	1.075	0.735	1.177	0.723
***f*_61_**	21 (4)	0.908	0.829	1.103	0.771
***f*_62_**	206 (8)	1.083	0.741	1.178	0.729
***f*_71_**	34 (4)	0.936	0.818	1.127	0.778
***f*_72_**	193 (8)	1.063	0.738	1.168	0.725
***f*_81_**	64 (6)	0.917	0.825	1.114	0.788
***f*_82_**	163 (8)	1.078	0.733	1.179	0.715
***f*_91_**	37 (4)	0.900	0.832	1.107	0.792
***f*_92_**	190 (8)	1.096	0.732	1.175	0.713

Note: ***f_i_*_1_**: PLS_GA_ model developed with the selected variables by GA method; ***f_i_*_2_**: PLS_RV_ model developed with the residual variables; ***i***: the i-th running the GA method. Letter superscript ^a^ is the number of spectral variables used for modeling, and ^b^ is the latent variables in PLS model.

## Data Availability

The data presented in this study are available on request from the corresponding author.

## References

[1] Niu Y., Deng J., Xiao Z., Zhu J. (2021). Characterization of the major aroma-active compounds in peach (*Prunus persica* L. Batsch) by gas chromatography–olfactometry, flame photometric detection and molecular sensory science approaches. Food Res. Int..

[2] Jaeger S.R., Axten L.G., Paisley A.G., Wohlers M.W., Marsh K.B., Sullivan M.B., Harker F.R. (2011). Developing models systems for testing the sensory properties and consumer acceptance of new fruit cultivars: The example of kiwifruit. Food Qual. Prefer..

[3] Lieb V.M., Esquivel P., Cubero Castillo E., Carle R., Steingass C.B. (2018). GC–MS profiling, descriptive sensory analysis, and consumer acceptance of Costa Rican papaya (*Carica papaya* L.) fruit purees. Food Chem..

[4] Xia Y., Xu Y., Li J., Zhang C., Fan S. (2019). Recent advances in emerging techniques for non-destructive detection of seed viability: A review. Artif. Intell. Agric..

[5] Grassi S., Alamprese C. (2018). Advances in NIR spectroscopy applied to process analytical technology in food industries. Curr. Opin. Food Sci..

[6] Zhang B., Gu B., Tian G., Zhou J., Huang J., Xiong Y. (2018). Challenges and solutions of optical-based nondestructive quality inspection for robotic fruit and vegetable grading systems: A technical review. Trends Food Sci. Technol..

[7] Olumegbon I.A., Oloyede A., Afara I.O. (2017). Near-infrared (NIR) spectroscopic evaluation of articular cartilage: A review of current and future trends. Appl. Spectrosc. Rev..

[8] Lohumi S., Lee S., Lee H., Cho B.-K. (2015). A review of vibrational spectroscopic techniques for the detection of food authenticity and adulteration. Trends Food Sci. Technol..

[9] Li M., Landahl S., East A.R., Verboven P., Terry L.A. (2019). Optical coherence tomography—A review of the opportunities and challenges for postharvest quality evaluation. Postharvest Biol. Technol..

[10] Porep J.U., Kammerer D.R., Carle R. (2015). On-line application of near infrared (NIR) spectroscopy in food production. Trends Food Sci. Technol..

[11] Yuan L.-M., Mao F., Chen X., Li L., Huang G. (2020). Non-invasive measurements of ‘Yunhe’ pears by vis-NIRS technology coupled with deviation fusion modeling approach. Postharvest Biol. Technol..

[12] Yuan L.-m., Sun L., Cai J.-r., Lin H. (2015). A Preliminary Study on Whether the Soluble Solid Content and Acidity of Oranges Predicted by Near Infrared Spectroscopy Meet the Sensory Degustation. J. Food Process Eng..

[13] Yuan L.-m., Cai J.-r., Sun L., Han E., Ernest T. (2016). Nondestructive Measurement of Soluble Solids Content in Apples by a Portable Fruit Analyzer. Food Anal. Methods.

[14] de Oliveira G.A., Bureau S., Renard C.M.-G.C., Pereira-Netto A.B., de Castilhos F. (2014). Comparison of NIRS approach for prediction of internal quality traits in three fruit species. Food Chem..

[15] de Araújo Gomes A., Azcarate S.M., Henrique Gonçalves Dias Diniz P., Douglas de Sousa Fernandes D., Veras G. (2021). Variable selection in the chemometric treatment of food data: A tutorial review. Food Chem..

[16] Xiaobo Z., Jiewen Z., Povey M.J.W., Holmes M., Hanpin M. (2010). Variables selection methods in near-infrared spectroscopy. Anal. Chim. Acta.

[17] Zareef M., Chen Q., Hassan M.M., Arslan M., Hashim M.M., Ahmad W., Kutsanedzie F.Y.H., Agyekum A.A. (2020). An Overview on the Applications of Typical Non-linear Algorithms Coupled With NIR Spectroscopy in Food Analysis. Food Eng. Rev..

[18] Vuolio T., Visuri V.-V., Sorsa A., Ollila S., Fabritius T. (2020). Application of a genetic algorithm based model selection algorithm for identification of carbide-based hot metal desulfurization. Appl. Soft Comput..

[19] Arakawa M., Yamashita Y., Funatsu K. (2011). Genetic algorithm-based wavelength selection method for spectral calibration. J. Chemom..

[20] Wang X., Feng H., Chen T., Zhao S., Zhang J., Zhang X. (2021). Gas sensor technologies and mathematical modelling for quality sensing in fruit and vegetable cold chains: A review. Trends Food Sci. Technol..

[21] Guo Z.-z., Chen X.-j., Yuan L.-m., Chen X., Zhu D.-h., Yang S. (2018). Consensus Modeling for Qualitative Analysis of Heavy Metal Cu in Tegillarca Granosa by LIBS Approach. Acta Photonica Sin..

[22] Yuan L.-m., Mao F., Huang G., Chen X., Wu D., Li S., Zhou X., Jiang Q., Lin D., He R. (2020). Models fused with successive CARS-PLS for measurement of the soluble solids content of Chinese bayberry by vis-NIRS technology. Postharvest Biol. Technol..

[23] Huang G., Ruan X., Chen X., Lin D., Liu W. (2014). A segmented PLS method based on genetic algorithm. Anal. Methods.

[24] Sheng R., Cheng W., Li H., Ali S., Akomeah Agyekum A., Chen Q. (2019). Model development for soluble solids and lycopene contents of cherry tomato at different temperatures using near-infrared spectroscopy. Postharvest Biol. Technol..

[25] Liu K., Chen X., Li L., Chen H., Ruan X., Liu W. (2015). A consensus successive projections algorithm—multiple linear regression method for analyzing near infrared spectra. Anal. Chim. Acta.

[26] Singh M., Singh R., Ross A. (2019). A comprehensive overview of biometric fusion. Inf. Fusion.

[27] Kiani S., Minaei S., Ghasemi-Varnamkhasti M. (2016). Fusion of artificial senses as a robust approach to food quality assessment. J. Food Eng..

[28] Nørgaard L., Saudland A., Wagner J., Nielsen J.P., Munck L., Engelsen S.B.J.A.s. (2000). Interval partial least-squares regression (i PLS): A comparative chemometric study with an example from near-infrared spectroscopy. Appl. Spectrosc..

[29] Guo W., Gu J., Liu D., Shang L. (2016). Peach variety identification using near-infrared diffuse reflectance spectroscopy. Comput. Electron. Agric..

[30] Nascimento P.A.M., Carvalho L.C.d., Júnior L.C.C., Pereira F.M.V., Teixeira G.H.d.A. (2016). Robust PLS models for soluble solids content and firmness determination in low chilling peach using near-infrared spectroscopy (NIR). Postharvest Biol. Technol..

[31] Nicolai B.M., Beullens K., Bobelyn E., Peirs A., Saeys W., Theron K.I., Lammertyn J. (2007). Nondestructive measurement of fruit and vegetable quality by means of NIR spectroscopy: A review. Postharvest Biol. Technol..

[32] Cen H., He Y. (2007). Theory and application of near infrared reflectance spectroscopy in determination of food quality. Trends Food Sci. Technol..

